# Quality of life after curative gastrectomy for gastric cancer in a randomised controlled trial

**DOI:** 10.1038/sj.bjc.6604097

**Published:** 2008-01-08

**Authors:** C-W Wu, J-M Chiou, F-S Ko, S-S Lo, J-H Chen, W-Y Lui, J Whang-Peng

**Affiliations:** 1 Department of Surgery, Taipei Veterans General Hospital, National Yang-Ming University, Taipei, Taiwan; 2 Institute of Statistical Science, Academia Sinica, Taipei, Taiwan; 3 Department of Research Resources, National Health Research Institutes, Taipei, Taiwan; 4 Institute of Cancer Research, National Health Research Institutes, Taipei, Taiwan

**Keywords:** quality of life, nodal dissection, gastric cancer, trial

## Abstract

Quality of life (QOL) was studied in gastric cancer patients treated on a randomised, controlled trial comparing D1 (level 1) with D3 (levels 1, 2 and 3) lymphadenectomy. A total of 221 patients were randomly assigned to D1 (*n*=110) and D3 (*n*=111) surgery. Quality-of-life assessments included functional outcomes (a 14-item survey about treatment-specific symptoms) and health perception (Spitzer QOL Index) was performed before and after surgery at disease-free status. Patients suffered from irrelative events such as loss of partners was excluded thereafter. Main analyses were done by intention-to-treat. Thus, 214 D1 (106/110=96.4%) and D3 (108/111=97.3%) R0 patients were assessed. Longitudinal analysis showed that functional outcomes decreased at 6 months after surgery and increased over time thereafter, while health perceptions increased over time in general. On the basis of linear mixed model analyses, patients having total gastrectomy, advanced cancer and hemipancreaticosplenectomy, but not complications had poorer QOL than those without. D1 and D3 patients showed no significant difference in QOL. The results suggest that changes of QOL were largely due to scope of gastric resection, disease status and distal pancreaticosplenectomy, rather than the extent of lymph node dissection. This indicates that nodal dissection can be performed for a potentially curable gastric cancer.

Gastric cancer remains the second most common cause of cancer-related deaths worldwide, and a major clinical challenge because of its poor prognosis and limited treatment options. The efficacy of nodal dissection is controversial (Cuschieri *et al*, 1999; Hartgrink *et al*, 2004). However, we have conducted a single institutional trial and demonstrated that D3 surgery has survival benefit (Wu *et al*, 2006b) but acceptable morbidity (Wu *et al*, 2004).

Quality of life (QOL) is looked upon as a multidimensional entity comprising physical, psychological, social and medical parameters. Cross-sectional studies (Koster *et al*, 1987; Kusche *et al*, 1987; Buhl *et al*, 1990; Korenaga *et al*, 1992; Jentschura *et al*, 1997; Wu *et al*, 1997a; Zieren *et al*, 1998; Svedlund *et al*, 1999; Thybush-Bernhardt *et al*, 1999) of QOL after gastric cancer surgery provided important background for design of this study, delineating a number of specific hypotheses and research questions related to surgery and survivorship. This report examines QOL of gastric cancer patients receiving either D1 or D3 surgery.

## MATERIALS AND METHODS

### Gastric cancer surgical trial summary

From October 1993 to August 1999, 221 patients were randomised at laparotomy to compare D1 (level 1) and D3 (level 1, 2 and 3) lymphadenectomy surgery for gastric cancer (Wu *et al*, 2004, 2006b). The primary surgical treatment report contains the full details. D3 surgery had more complications than D1 surgery, but both had no deaths (Wu *et al*, 2004, 2006a). No patients received preoperative or postoperative adjuvant radiotherapy or chemotherapy. D3 surgery offers a survival benefit when done by well-trained, experienced surgeons (Wu *et al*, 2006b).

### Eligibility for the QOL study

The QOL study, a companion protocol, was opened simultaneously with surgical trial. Patients were eligible if they were registered to the National Health Research Institutes and had fitted the requirement of operation criteria and randomisation. The exclusion criteria included patients not curatively resected and not treated according to protocol. All patients were at disease-free status when the survey was carried out, because tumour recurrence was the decisive exclusion factor. The Ethics Committee approved the study protocol and an informed consent was obtained from each patient.

### Instruments, assessment strategy and procedures

The Spitzer QOL Index (Spitzer *et al*, 1981), a global health assessment with valid questionnaire, includes five items rated on a three-point scale: activity, daily living, health, support of family and friends, and outlook. Functional outcome was assessed using a 14-item survey designed to assess treatment-specific symptoms, largely gastrointestinal function. Low scores reflect more symptoms (Korenaga *et al*, 1992; Wu *et al*, 1997a). Its validity was checked by the Cronbach’s *α*-scores.

The assessment strategy focused on QOL at a disease-free status not to be confounded by irrelevant events such as loss of partners and bony fractures after falling. We hypothesised that patients receiving D3 resection would have more symptoms and greater fatigue than patients receiving D1 surgery, with treatment arm differences most prominent at the 6-month assessment, and possibly continuing up to 1 year after random assignment. Questionnaires were administered at registration, 6 months, 1 year and annually thereafter until recurrence of tumour, if any. A research nurse (Hsueh-Pin Yu) briefly described and explained the procedure for responding to each question. Each interview took approximately 20 min.

### Statistical considerations and analytic plan

All analyses used the intention-to-treat philosophy, whereby patients were grouped according to randomly assigned surgery treatment arms and under the assumption of a missing-at-random mechanism. The lymph node dissection effects on the functional outcome, and Spitzer QOL Index were examined by the linear mixed models (Diggle *et al*, 2002), adjusting for fixed effects for each time point, treatment, baseline covariate, age, sex, marital status, scope of gastric resection and gross appearance of tumour, and with random effects for intercept and each time point. Quadratic times as well as interaction terms were considered in the models to adjust for nonlinear temporal trend. A general unstructured covariance was assumed, that is, no specific forms of the random-effects covariance matrix were assumed. The lymph node dissection effects were assessed based on *P*-values of the fitted coefficients, adjusting for significant covariates in the model. The time trends of the functional outcome and Spitzer Index were assessed by significance tests of adjacent time periods in the mixed models, assisted by the longitudinal plots classified by the D1 and D3 treatment groups. These models and analyses were implemented by SAS PROC Mixed procedure.

## RESULTS

### Patient characteristics

In all, 214 R0 patients (mean age 63.9±11.1 years) were recruited. Comparison of the QOL study sample with the final surgical trial sample demonstrated no statistical difference between D1 (106/110=96.4%) and D3 (108/111=97.3%) patients in the QOL study (*P*=0.772, *χ*^2^ test). Male patients were older (65.43±10.3 *vs* 58.8±12.2) and underwent more distal pancreaticosplenectomy procedures. Female patients as compared with male patients were more likely to be married (100 *vs* 90.8%). The two groups did not differ significantly according to age, gender, marital status, type of gastric resection, reconstructive procedures (Table 1), comorbidity and location of tumour. But D3 patients had more cholecystectomy, distal hemipancreaticosplenectomy procedures (Table 1) and complications. During follow-up, five patients encountered irrelevant events; two spouse suicides (fifth year), three bony fractures (at second, second and sixth years, respectively). Four patients immigrated to Mainland China (at second, second, third and third years, respectively) and therefore could not be assessed at regular time points thereafter. Quality of life was not assessed at scheduled across assessment points after surgery in 3.2% of the subjects due to prolonged absence overseas. The Cronbach’s *α*-scores for treatment-specific symptoms were 0.67.

### Longitudinal analysis of QOL

**Figure 1:**
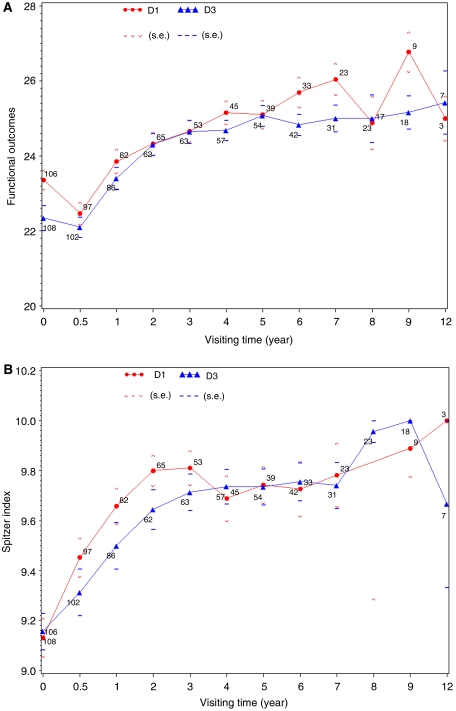
(**A**) Average total evaluation score for functional outcome (treatment-specific symptoms), adjusted for time trend and baseline score by treatment arms. Mean scores are presented with s.e. (bars). High scores reflect better quality of life. (**B**) Average total evaluation score for general health perception (Spitzer Index) adjusted for time trend and baseline score by treatment arms. Mean scores are presented with s.e. (bars). High scores reflect better perception of health status.

Nodal dissection effect showed transient discrepancy of food volume at baseline, diarrhoea and heartburn at first and second years after surgery (Table 2). The linear mixed model analysis for functional outcome showed that there were no significant differences in scores between two surgical treatment groups (*P*=0.5338, 0.6423 and 0.3941 for nodal dissection effect and its interaction effects with time and time^2^, respectively), after adjusting for significant covariate effects (Table 3). These significant covariate effects included time trend, baseline score, age, gender, gross appearance of tumour, scope of gastric resection and hemipancreaticosplenectomy. Surgical complications, depth of cancer invasion and marital status did not affect functional outcomes, and therefore were dropped from the model. These results coincide with univariate analyses (which were not shown here) based on linear mixed effects models when a single covariate effect is considered, adjusted for time trends and baseline scores. The overall time trends (Figure 1) indicated that the functional outcomes were significantly lower at the 6th month after surgical treatment than at the baseline (*P*<0.0001), and improved from the sixth month to the first year after surgery (*P*<0.0001), continuously improving up to the second year after surgery (*P*=0.006). Thereafter, the differences in functional outcomes between adjacent years were not statistically significant. The time trends of functional outcomes had to be adjusted by significant interaction effects with time, including scope of gastric resection, gross appearance of tumour, age and baseline score.

For the Sptizer Index scores, the linear mixed model analysis revealed similar results that there were no significant differences in scores between two surgical treatment groups (*P*=0.5652, 0.2956 and 0.0784 for node dissection effect and its interaction effects with time and time^2^, respectively), after adjusting for significant covariate effects (Table 3). The significant covariate effects are similar to those on the functional outcomes. An interesting finding is that the marital status is a significant effect on the Sptizer Index scores, but not on the functional outcomes. The overall health perceptions (Spitzer Index) (Figure 1) were significantly lower at the baseline than at the 6th month after surgical treatment (*P*<0.0001), and improved from the 6th month to the first year after surgery (*P*=0.0022). Thereafter, there were no significant differences in health perceptions between adjacent years. Random effects are included in the statistical analysis, but not reported in Table 3.

Further analysis based on overall averaged scores showed that patients who underwent a distal pancreatic resection had poorer appetite (*P*=0.001 based on *t*-test), decreased consistency (*P*<0.0001) and volume of food (*P*=0.0003) and frequency of eating (*P*<0.0001), lost of more body weight (*P*=0.012), could not readily engage in full-time work or study (*P*<0.0001), lacked energy (*P*<0.0001) and reported less psychosocial support (*P*=0.021) than those without pancreatic resection.

## DISCUSSION

The results of the longitudinal QOL measurements indicate that D1 and D3 gastric cancer patients have similar health perception and functional outcomes after surgery.

When we designed QOL study the Functional Assessment of Cancer Therapy–General (FACT-G) (Cella *et al*, 1993) and the European Organization for Research and Treatment of Cancer Core Quality of Life Questionnaire (EORTC QLQ-C30) (Aaronson *et al*, 1993) were not available, and no Chinese version was available. The Spitzer QOL Index is a valid but suboptional questionnaire due to the limited number of items and ceiling effect. However, it is easily applied and can be summed into a single score while retaining its domain-specific properties. It has been used worldwide in various cancers (Greimel *et al*, 2002; Erridge *et al*, 2005) including gastric cancer (Kusche *et al*, 1987; Buhl *et al*, 1990). Our treatment-specific instrument, which was modified from Korenaga's study for Japanese gastric cancer patients, is a short questionnaire, which has proved to be reliable in implementation (Wu *et al*, 1997a, 2000) and in current study (Cronbach's *α*-scores 0.67). Taken all questionnaires together, data acquisition for 19 items assisted by a research nurse had shown its validity throughout the entire study.

Patients had anxiety, depression, loss of appetite and body weight at diagnosed of gastric cancer, but returned to normal gradually after surgery. Similar to other observations (Jentschura *et al*, 1997; Wu *et al*, 1997a; Davies *et al*, 1998), patients after a total gastrectomy tended to suffer poorer tolerance of normal food, need for more frequent eating and loss of more body weight than those after a subtotal gastrectomy. And younger patients had better QOL than older (Koster *et al*, 1987; Wu *et al*, 1997a). We noted that gender plays an important role in facing gastric cancer. Male patients usually have more morbidity after gastric cancer surgery (Viste *et al*, 1988). It is noteworthy that female patients were younger and mostly married, but male patients tended to feel better and calmer, suffer less insomnia and have a more positive outlook. This may not be simply explained by different culture roles attributes for men and women, since Koster *et al* (1987) also recorded the same phenomenon in German gastric cancer patients.

Lymph node dissection along the upper border of the pancreas is essential for radical gastric cancer surgery. Resection of distal pancreas to facilitate lymph node dissection had a high morbidity and mortality. (Bonenkamp *et al*, 1995; Cuschieri *et al*, 1996; Wu *et al*, 2006a) Our data revealed that diarrhoea, except at first year, was similarly experienced in both groups. This study further disclosed its worse functional outcome and health perception, but no negative effect of QOL among D3 patients was drawn as the number of affected patients (1% in D1 *vs* 12% in D3 patients) was small. However, a technique developed by Maruyama *et al* (1995) to preserve the pancreas is feasible and safe, and should be considered in gastric cancer surgery.

Although the morbidity rate was higher in D3 patients than in D1 patients, our analysis indicates that lymph node dissection did not adversely influence QOL. The possible explanations are that the anastomotic leaks were minor in extent and abscess was detected early. These patients had no fever and did not need intensive care. They consumed regular diet while treating by a closed continuous irrigation system for abscess, and leaks were managed nonoperatively with nutritional support (Wu *et al*, 2004) Nevertheless, one should be very cautious in interpreting these results because the postoperative morbidity rate was very low, as compared with other reported studies (Bonenkamp *et al*, 1995; Cuschieri *et al*, 1996). Our previous study and current trial showed that operative morbidity did not influence survival (Wu *et al*, 1997b, 2006b). The findings we observed are apparently at variance with those of a previous study (Siewert *et al*, 1998). A similar disparity also exists in QOL.

## Figures and Tables

**Table 1:** Patient and operation characteristics

	**D1 (*n*=106)**	**D3 (*n*=108)**	***P***
*Age (years)*			
Mean (95%)	62.8 (60.6–64.9)	65.0 (62.9–67.0)	0.149
			
*Sex*			
Men	80	83	
Women	26	25	0.813
			
*Marital status*			
Yes	100	99	
No	6	9	0.444
			
*Type of gastric resection*			
Total gastrectomy	29	22	
Distal subtotal gastrectomy	77	86	0.230
			
*Combined resection*			
Cholecystectomy	14	38	<0.001
Distal pancreaticosplenectomy	1	13	0.001
Segmental resection of colon	3	0	0.120
Splenectomy	3	1	0.015
Partial liver resection	2	1	0.620
Oophorectomy (for ovarian cyst)	1	0	0.495
			
*Reconstructive procedures*			
Billroth I	6	8	
Billroth II	71	78	
Roux-en-Y	29	22	0.459

**Table 2:** Effect of nodal dissection on quality of life

	**Time after operation**																				
	**Baseline**			**Half year**			**First year**			**Second year**			**Third year**			**Fourth year**			**Fifth year**		
**Disease-specific symptom**	**D1 (*n*=106)**	**D3 (*n*=108)**	***P*-value**	**D1 (*n*=97)**	**D3 (*n*=102)**	***P*-value**	**D1 (*n*=82)**	**D3 (*n*=86)**	***P*-value**	**D1 (*n*=65)**	**D3 (*n*=62)**	***P*-value**	**D1 (*n*=53)**	**D3 (*n*=63)**	***P*-value**	**D1 (*n*=45)**	**D3 (*n*=57)**	***P*-value**	**D1 (*n*=39)**	**D3 (*n*=54)**	***P*-value**
*Treatment-specific symptoms*																					
Appetite	1.03 (0.58)	0.91 (0.59)	0.13	1.15 (0.46)	1.15 (0.55)	0.92	1.35 (0.55)	1.34 (0.52)	0.84	1.45 (0.53)	1.44 (0.53)	0.91	1.49 (0.50)	1.48 (0.50)	0.88	1.64 (0.48)	1.51 (0.50)	0.17	1.49 (0.51)	1.54 (0.50)	0.64
Consistency of food	1.82 (0.41)	1.82 (0.41)	0.95	1.79 (0.41)	1.83 (0.37)	0.48	1.93 (0.26)	1.92 (0.31)	0.85	1.94 (0.24)	1.97 (0.18)	0.44	2.00 (0.00)	2.00 (0.00)	—	2.00 (0.00)	1.98 (0.13)	0.38	1.95 (0.22)	2.00 (0.00)	0.09
Volume of food	0.80 (0.42)	0.68 (0.47)	0.04^*^	0.48 (0.58)	0.40 (0.58)	0.32	0.87 (0.60)	0.80 (0.61)	0.50	0.95 (0.60)	0.94 (0.62)	0.87	1.00 (0.44)	1.02 (0.49)	0.86	1.07 (0.45)	1.09 (0.39)	0.80	1.26 (0.44)	1.26 (0.44)	0.98
Frequency of eating	1.96 (0.19)	1.94 (0.27)	0.58	1.12 (0.53)	1.21 (0.55)	0.28	1.44 (0.55)	1.51 (0.53)	0.38	1.69 (0.50)	1.66 (0.48)	0.72	1.91 (0.30)	1.84 (0.37)	0.31	1.96 (0.21)	1.96 (0.19)	0.81	1.97 (0.16)	1.98 (0.14)	0.82
Eating time	1.96 (0.19)	1.95 (0.21)	0.76	1.99 (0.10)	1.97 (0.17)	0.34	2.00 (0.00)	2.00 (0.00)	—	1.98 (0.12)	1.98 (0.13)	0.97	2.00 (0.00)	2.00 (0.00)	—	2.00 (0.00)	2.00 (0.00)	—	2.00 (0.00)	2.00 (0.00)	—
Postprandial abdominal fullness	1.19 (0.52)	1.17 (0.56)	0.82	1.41 (0.54)	1.34 (0.47)	0.29	1.50 (0.50)	1.48 (0.50)	0.76	1.54 (0.50)	1.52 (0.50)	0.80	1.47 (0.50)	1.56 (0.50)	0.37	1.49 (0.51)	1.35 (0.48)	0.16	1.54 (0.51)	1.46 (0.50)	0.48
Heartburn	1.71 (0.50)	1.62 (0.59)	0.24	1.95 (0.22)	1.97 (0.17)	0.43	1.95 (0.27)	1.94 (0.24)	0.81	1.88 (0.38)	1.98 (0.13)	0.04^*^	1.92 (0.27)	1.97 (0.18)	0.29	1.98 (0.15)	1.93 (0.26)	0.27	1.95 (0.22)	1.91 (0.29)	0.46
Diarrhoea	1.81 (0.44)	1.73 (0.54)	0.24	1.82 (0.38)	1.72 (0.47)	0.08	1.88 (0.36)	1.69 (0.51)	0.01^*^	1.80 (0.40)	1.74 (0.44)	0.44	1.81 (0.39)	1.76 (0.50)	0.56	1.84 (0.37)	1.74 (0.44)	0.19	1.79 (0.47)	1.76 (0.47)	0.72
Constipation	1.78 (0.41)	1.71 (0.53)	0.30	1.85 (0.42)	1.82 (0.43)	0.70	1.82 (0.42)	1.85 (0.39)	0.61	1.88 (0.38)	1.77 (0.46)	0.17	1.87 (0.34)	1.71 (0.49)	0.06	1.62 (0.53)	1.72 (0.45)	0.32	1.64 (0.54)	1.69 (0.47)	0.67
Insomnia	1.88 (0.41)	1.90 (0.34)	0.73	1.86 (0.43)	1.89 (0.31)	0.51	1.84 (0.48)	1.81 (0.45)	0.70	1.85 (0.44)	1.92 (0.27)	0.27	1.83 (0.43)	1.84 (0.41)	0.89	1.82 (0.44)	1.91 (0.29)	0.22	1.79 (0.41)	1.87 (0.34)	0.33
Body weight	1.95 (0.74)	1.81 (0.73)	0.14	1.56 (1.00)	1.38 (0.98)	0.21	1.60 (1.04)	1.50 (1.06)	0.55	1.62 (1.00)	1.69 (0.92)	0.65	1.64 (0.90)	1.75 (0.93)	0.54	1.89 (0.91)	1.77 (0.91)	0.52	1.87 (0.98)	1.85 (0.96)	0.92
Swallowing problem	1.96 (0.19)	1.98 (0.14)	0.42	1.97 (0.23)	1.98 (0.14)	0.68	2.00 (0.00)	1.99 (0.11)	0.33	1.98 (0.12)	1.98 (0.13)	0.97	2.00 (0.00)	2.00 (0.00)	—	2.00 (0.00)	2.00 (0.00)	—	2.00 (0.00)	2.00 (0.00)	—
Vomiting	1.81 (0.44)	1.70 (0.57)	0.10	1.97 (0.17)	1.93 (0.26)	0.22	1.96 (0.19)	1.97 (0.18)	0.95	1.98 (0.12)	1.98 (0.13)	0.97	2.00 (0.00)	2.00 (0.00)	—	2.00 (0.00)	1.98 (0.13)	0.38	2.00 (0.00)	2.00 (0.00)	—
Dizziness	1.69 (0.54)	1.70 (0.52)	0.83	1.54 (0.52)	1.61 (0.49)	0.28	1.72 (0.55)	1.62 (0.49)	0.20	1.78 (0.41)	1.73 (0.45)	0.44	1.72 (0.45)	1.73 (0.45)	0.88	1.84 (0.37)	1.74 (0.44)	0.19	1.85 (0.37)	1.74 (0.44)	0.23
																					
*Spitzer Index*																					
Activity	1.93 (0.25)	1.95 (0.21)	0.53	1.97 (0.17)	1.94 (0.24)	0.35	1.99 (0.11)	1.95 (0.21)	0.19	2.00 (0.00)	1.98 (0.13)	0.31	1.98 (0.14)	1.98 (0.13)	0.90	2.00 (0.00)	1.98 (0.13)	0.38	2.00 (0.00)	2.02 (0.14)	0.40
Daily living	1.99 (0.10)	1.99 (0.10)	0.99	2.00 (0.00)	1.98 (0.14)	0.17	2.00 (0.00)	2.00 (0.00)	—	1.98 (0.12)	1.98 (0.13)	0.97	1.98 (0.14)	2.00 (0.00)	0.28	2.00 (0.00)	2.00 (0.00)	—	2.00 (0.00)	2.00 (0.00)	—
Health	1.70 (0.46)	1.68 (0.47)	0.73	1.72 (0.45)	1.68 (0.47)	0.49	1.84 (0.37)	1.79 (0.41)	0.40	1.89 (0.31)	1.85 (0.36)	0.53	1.94 (0.23)	1.86 (0.35)	0.13	1.87 (0.34)	1.88 (0.33)	0.88	1.87 (0.34)	1.87 (0.34)	0.98
Support	1.93 (0.25)	1.93 (0.26)	0.82	1.99 (0.10)	1.95 (0.22)	0.11	1.99 (0.11)	1.95 (0.21)	0.19	2.00 (0.00)	1.95 (0.22)	0.07	1.98 (0.14)	1.98 (0.13)	0.90	1.93 (0.25)	1.96 (0.19)	0.47	1.95 (0.22)	1.96 (0.19)	0.74
Outlook	1.58 (0.50)	1.61 (0.49)	0.60	1.77 (0.42)	1.76 (0.43)	0.89	1.84 (0.37)	1.80 (0.40)	0.51	1.92 (0.27)	1.87 (0.34)	0.34	1.92 (0.27)	1.89 (0.32)	0.52	1.89 (0.32)	1.91 (0.29)	0.70	1.92 (0.27)	1.91 (0.29)	0.79

^*^Statistically significant.

**Table 3:** Estimates of regression coefficients by linear mixed model for functional outcomes and health perception (Spitzer Index) scores

	**Estimate**	**s.e.**	**P>F**
*Effect on functional outcomes*			
Intercept	13.4639	1.6958	<0.0001
Time	5.0898	0.9718	<0.0001
Time^2^	−0.4664	0.1229	0.0002
Node dissection (D3)	−0.2386	0.3832	0.5338
Node dissection*time	0.0825	0.1776	0.6423
Node dissection*time^2^	−0.0190	0.0223	0.3941
Scope of gastrectomy (subtotal)	2.0668	0.4825	<0.0001
Scope of gastrectomy*time	−0.5924	0.2276	0.0095
Scope of gastrectomy*time^2^	0.0616	0.0295	0.0368
Gender (male)	1.6467	0.3255	<0.0001
Gross appearance (Borrmann III and IV)	−0.7142	0.3381	0.0352
Gross appearance*time	0.1696	0.0594	0.0044
Age*time	−0.0175	0.0066	0.0079
Age*time^2^	0.0011	0.0009	0.2284
Hemipancreaticosplenectomy (yes)	−1.4653	0.6907	0.0344
Baseline	0.2834	0.0670	<0.0001
Baseline*time	−0.1189	0.0333	0.0004
Baseline*time^2^	0.0124	0.0041	0.0021
			
*Effect on health perception (Spitzer Index) scores*			
Intercept	8.0906	0.3663	<0.0001
Time	0.1879	0.0475	0.0001
Time^2^	−0.0254	0.0065	0.0002
Node dissection (D3)	−0.0547	0.0951	0.5652
Node dissection*time	−0.0662	0.0633	0.2956
Node dissection*time^2^	0.0150	0.0085	0.0784
Scope of gastrectomy (subtotal)	0.1074	0.0655	0.1016
Gender (male)	0.2281	0.0585	0.0001
Gross appearance (Borrmann III and IV)	−0.1361	0.0479	0.0047
Age	−0.0056	0.0023	0.0137
Marital status (married)	0.2642	0.0929	0.0047
Hemipancreaticosplenectomy (yes)	−0.6687	0.1638	<0.0001
Hemipancreaticosplenectomy*time	0.1346	0.0598	0.0250
Baseline	0.1396	0.0321	<0.0001
